# A new biological species in the *Mercurialis annua* polyploid complex: functional divergence in inflorescence morphology and hybrid sterility

**DOI:** 10.1093/aob/mcz058

**Published:** 2019-05-17

**Authors:** Wen-Juan Ma, Luis Santos del Blanco, John R Pannell

**Affiliations:** Department of Ecology and Evolution, University of Lausanne, Lausanne, Switzerland

**Keywords:** Male-like inflorescence, polyploidy, functional divergence, female sterility, reproductive isolation, phylogeny, evolutionary origin

## Abstract

**Background and Aims:**

Polyploidy has played a major role in the origin of new plant species, probably because of the expansion of polyploid populations in the species’ ecological niche, and because reproductive isolation can be established between a new polyploid population and its diploid progenitor species. It is well established that most polyploid species are polyphyletic, with multiple independent origins, and that polyploid genomes may undergo rapid change after their duplication and hybridization associated with their origin. We considered whether multiple independent origins and rapid genomic change might lead to reproductive isolation between polyploid populations of the same ploidy but with potentially different evolutionary histories.

**Methods:**

We tested our hypothesis by assessing differences in DNA content and morphology, the evolution of reproductive isolation, and the phylogenetic placement of two broadly sympatric hexaploid lineages of the wind-pollinated annual plant *Mercurialis annua* hitherto regarded as populations of the same species.

**Key Results:**

The two hexaploid lineages of *M. annua* have slightly divergent DNA content, and distinct inflorescence morphology. They also fall into largely different clades of a chloroplast phylogeny and are reproductively isolated from one another.

**Conclusions:**

The distinct evolutionary histories of the two hexaploid lineages of *M. annua* have contributed to the remarkable reproductive diversity of the species complex. It seems likely that reproductive interference between them will eventually lead to the displacement of one lineage by the other via pollen swamping. Thus, whereas polyploidization can contribute to speciation, diversification might also be compromised by reproductive interference.

## INTRODUCTION

Whole genome duplication and polyploidy have played major roles in the diversification of land plants (Stebbins, 1950; Levin, 1983; Soltis *et al.*, 2014a, b). Recent polyploids account for about 35 % of flowering plant species (Wood *et al.*, 2009), and probably all angiosperm lineages have experienced polyploidy in their distant past (Soltis *et al.*, 2009). The success of polyploids, particularly allopolyploids, is thought to be due to the genomic flexibility and increased heterozygosity that results from the combination of two or more genomes, conferring on individuals an ability to colonize a broader range of environments than their diploid progenitors (Soltis *et al.*, 2004, 2014; Hegarty and Hiscock, 2008; but see Glennon *et al.*, 2014). Polyploidization may also contribute to diversification through the reciprocal loss of duplicated genes, as shown in yeast and *Arabidopsis* (Scannell *et al.*, 2006, 2007*a, b*; Bikard *et al.*, 2009). Yet if polyploid lineages were the result of a unique event of hybridization and genome duplication, as they were thought to be (Otto, 2007), the adaptive flexibility of having multiple genomes should be limited by the narrow genetic bottleneck of their origin. However, it has been clear for more than two decades that most polyploid species are polyphyletic, i.e. the result of multiple, independent episodes of hybridization and genome duplication (Soltis and Soltis, 1999). The polyphyletic origin of polyploid species probably contributes substantially to their commonly high genetic diversity, ecological breadth and evolutionary potential (Lewis, 1980; Pannell *et al.*, 2004; Soltis *et al.*, 2004, 2014a; Beest *et al.*, 2012).

The high genetic diversity observed in polyploid species may also be due to an accelerated rate of genome reorganization and other mutational processes that follow hybridization and genome duplication. It is not straightforward to characterize such processes in long-established polyploid species, but analysis of the immediate descendants of synthetic polyploids has revealed that these processes can be dramatic. For instance, Song *et al.* (1995) observed changes in restriction fragment patterns in each of the first few generations after synthesizing polyploids from several *Brassica* species, attributing these changes to chromosomal rearrangements, point mutations, gene conversion and DNA methylation. Subsequent research indicates that accelerated evolution of new polyploid lineages is probably frequent (Chen and Ni, 2006; Chen *et al.*, 2007), but not ubiquitous (e.g. Liu *et al.*, 2001). A revealing example is provided by the high cytological and morphological variation observed in the arctic polyploid species *Saxifraga cernua*, this variation probably having arisen multiple times (Brochmann *et al.*, 1998).

The polyphyletic origin and rapid genome evolution of polyploid lineages should not only contribute to their ecological breadth, but also to their potential for subsequent diversification. Substantial attention has been paid to establishing the extent and implications of reproductive isolation between polyploids and their diploid relatives, through both experimental crosses (Maceira *et al.*, 1993; Husband *et al.*, 2002; Buggs and Pannell, 2006) and genome analysis (Song *et al.*, 1995; Soltis and Soltis, 2000; Chen and Ni, 2006). Although reproductive isolation between polyploids and their diploid progenitors as a result of their differing chromosome numbers may be an important engine of diversification (Thompson and Lumaret, 1992; Ramsey and Schemske, 1998; Soltis and Soltis, 2009; but see Baduel *et al.*, 2018 for a discussion of evidence for cross-ploidy gene flow), different independent origins of the same polyploid taxon might also contribute to diversification if they are reproductively isolated from one another, particularly if they have been subject to rapid evolutionary change following hybridization and/or genome duplication. Yet there appears to be an almost complete lack of evidence for or against the hypothesis that polyploid populations of the same ploidy levels with different origins might be reproductively isolated. Combined with the potentially accelerated evolution of new polyploid lineages due to genome reorganization (Song *et al.*, 1995; Soltis and Soltis, 2000) and changes in gene expression (Chen and Ni, 2006), such reproductive isolation would evidently have important taxonomic and ecological implications.

In the present study, we reveal that broadly sympatric hexaploid monoecious populations of the European wind-pollinated annual plant *Mercurialis annua* (Euphorbiaceae) belong to at least two different lineages that are strongly reproductively isolated from one another, and that probably had an independent origin. Our study was stimulated by the observation of morphological differences in inflorescence architecture among populations and an interest in the functional significance of that variation. The *M. annua* species complex displays remarkable variation in its sexual systems, including dioecy, monoecy and the rare sexual system androdioecy, where males co-occur with monoecious hermaphrodites (Pannell, 1997*a*, 2008; Obbard *et al.*, 2006a,b; Russell and Pannell, 2015). Against this background, variation in inflorescence architecture among monoecious populations allows valuable comparisons among divergent inflorescence strategies for outcross siring success (Santos del Blanco *et al.*, 2019), but it also prompts questions concerning its origins.

**Fig. 1.**
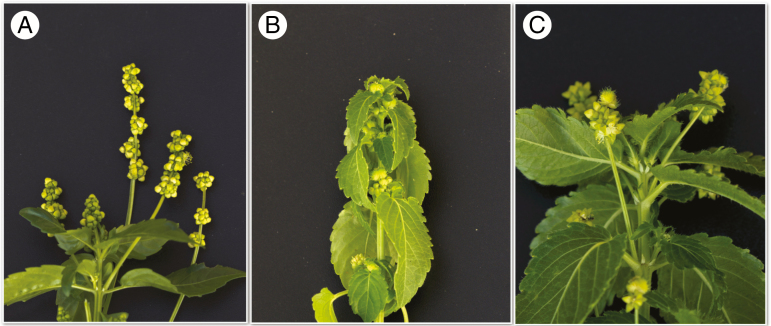
Images of three different sexual phenotypes of hexaploid *Mercurialis annua*: (A) a male individual, showing flowers on peduncles; (B) a P− monoecious individual, with male and female flowers held in leaf axils; and (C) a P+ monoecious individual, with flowers held on erect inflorescence stalks, or ‘peduncles’. Images courtesy of Xinji Li.

In the *M. annua* species complex, diploid populations are dioecious, but hexaploid populations are either monoecious or androdioecious (Obbard *et al.*, 2006a,b). Diploid and polyploid males hold their flowers in erect ‘pedunculate’ inflorescences above the plant (Fig. 1A), whereas diploid females and hexaploid monoecious individuals (which are effectively modified pollen-producing females) typically hold both their male and their female flowers in sub-sessile inflorescences in the leaf axils (Hesse and Pannell, 2011*a*, *b*: Fig. 1B). However, we recently documented the existence in some populations of apparently hexaploid monoecious individuals of *M. annua* that, like males, hold virtually all their male flowers on erect peduncles rather than in the sub-sessile axillary inflorescences that are more typical of hexaploid *M. annua*, whereas female flowers are found on short pedicels in the leaf axils or, occasionally, on the erect peduncles along with male flowers (Fig. 1C; Santos del Blanco *et al.*, 2019). The non-pedunculate form of hexaploid monoecious *M. annua* (which we here label ‘P–‘) occurs widely around the Iberian Peninsula in disturbed ruderal habitats, often together with males in androdioecious populations (Pannell, 1997*a*; Buggs and Pannell, 2006; Obbard *et al.*, 2006a). The pedunculate phenotype (labelled ‘P+’), although broadly sympatric with the P– phenotype along the Spanish Mediterranean coastal areas and occupying very similar habitats, occurs rarely in mixed populations with the P– phenotype. A previous study found that the P+ phenotype is substantially better at dispersing pollen than the P– phenotype and enjoys much greater siring success in experimental populations, without any obvious phenotypic advantage to the P– phenotype (Santos del Blanco *et al.*, 2019). One might expect such a superior strategy for pollen dispersal to spread quickly throughout the species range, particularly as it does not appear very costly in terms of other fitness components (Buggs and Pannell, 2006). However, the results we present here indicate that the P– and P+ phenotypes, which are both hexaploid and broadly sympatric, nonetheless belong to two different biological species.

We characterized the variation between the P– and P+ phenotypes of hexaploid *M. annua* in terms of inflorescence and vegetative morphology, ploidy and DNA quantity, their position in a phylogeny of the genus based on both nuclear and chloroplast markers (Obbard *et al.*, 2006a), and experimental crosses. Although the two phenotypes are morphologically and ecologically very similar, all our data are consistent with the hypothesis that they belong to two different evolutionary lineages. Our study shows that the *M. annua* species complex is even more diverse than hitherto thought. The finding of reproductive isolation between two polyploid lineages with the same ploidy level but apparently different hybridization pathways points to a potentially important but underexplored mode of diversification in flowering plants. It also raises interesting questions concerning the long-term evolutionary maintenance of distinct lineages that occupy similar habitats and are susceptible to strongly detrimental effects of reproductive interference through the generation of sterile hybrid progeny.

## MATERIALS AND METHODS

### Source populations

We sampled plants of hexaploid *M. annua* from a total of 26 source populations (Supplementary Data Table S1) in 2013 in eastern Spain, in the region where populations of P– and P+ individuals broadly co-occur (Supplementary Data Fig. S1). Seeds were pooled from ~30 individuals from each of 13 populations with P– and 13 populations with P+ individuals (Fig. 1). Plants were raised from these seeds under uniform conditions in a glasshouse at the University of Lausanne, Switzerland, and their seed progeny were harvested for use in various experiments as detailed below.

### Common garden experiment and morphological trait measurements

We recorded morphological trait values for individuals from 21 populations of *M. annua* in our common garden experiment. Seeds were first sown in bulk in seedling trays, and seedlings were then transplanted individually into 15-cm pots ~2 weeks after emergence. Pots were arranged in a complete randomized block design, with 11 blocks and 21 pots per block, i.e. one per population. Plants were watered as needed and grown under uniform glasshouse conditions (23–25 °C, 50 % humidity, 12: 12 light–dark cycle).

Plants were harvested 7 weeks after transplanting, and the following morphological traits were measured and recorded: plant height; length of one of each of the pairs of branches emanating from the first three basal nodes (branches 0, 1 and 2); length of third, fourth and fifth internodes; total number of male peduncles on the plant; length and number of male flowers on five randomly chosen male peduncles; number of peduncles with fruits instead of male flowers at their apex; number of peduncles protruding above the plant’s uppermost leaves; and the biomass of all male flowers on the plant (following their removal and drying at 40 °C for 1 week). For one leaf emanating from each of the third, fourth and fifth internodes on the main shoot, we also estimated the leaf blade area, leaf blade circularity, leaf blade length, leaf blade perimeter, leaf blade width, petiole length and leaf perimeter area, based on scanning and image analysis using the software LeafJ (Maloof *et al.*, 2013). We also recorded whether the leaves had curly margins or not, as well as the presence or absence of trichomes on stems. Finally, we recorded total male flower biomass, total seed biomass and total above-ground plant biomass for each plant. Seed number and the size distribution of seeds were obtained with an Elmor C3 seed counter (SA. Ltd, Schwyz, Switzerland). Male and female reproductive effort was estimated as male-flower and seed biomass, respectively, divided by above-ground plant biomass. An index of sex allocation was estimated as male-flower biomass divided by the total biomass of pollen and seeds together.

We used principal component analysis (PCA) based on all traits to characterize the overall morphological differences between the two phenotypes. We used generalized linear mixed models (GLMMs) using the lme4 package in R version 3.4.3 (R Core Team, 2017) to assess differences in morphology between the two phenotypes for specific traits, with phenotype defined as a fixed effect, and population and block defined as random effects. Gaussian models were used for all variables, except for the presence or absence of trichomes or curly leaves, which were analysed with binomial models.

### Genome size estimation

We used flow cytometry to estimate the genome size and ploidy level of three to five individuals from each population of P– and P+ phenotypes, modifying the protocols described by Obbard *et al.* (2006a) and Ma *et al.* (2013). Briefly, we chopped a mix of 10 mg of leaf material from *M. annua* with 5 mg of a fresh mature leaf of tomato (*Solanum lycopersicum*) as an internal control in 1000 µL ice-cold Galbraith buffer (Galbraith *et al.*, 1983). We strained the mix through a 35-μm membrane filter of Falcon tubes (12 × 75 mm), and stained the strained material with 60 µL DAPI (0.02 µL mL^–1^, 1.2 µL). The stained DNA samples were mixed in a gentle vortex, and then kept at 4 °C for ~30 min before analysis with a flow cytometer (BD LSRFortessa, BD Biosciences, Franklin Lakes, NJ, USA). Data were analysed using the software BDFACSDiva, with the combination of populations from the same and other ploidy levels of *M. annua*, as described and analysed by Obbard *et al.* (2006a). Finally, the target genome size (pg) was calculated as (mean fluorescence target/mean fluorescence control) × genome control (Hare and Johnston, 2012). We used a GLM with the multicomp function in R version 3.4.3 (R Core Team, 2017) to compare the genome size among the sampled populations.

### Crossing array for the production of hybrid and non-hybrid progeny

We conducted reciprocal crosses between sets of P– and P+ monoecious individuals to assess evidence for post-zygotic reproductive isolation, choosing two populations of the P– phenotype (P-GAU, P-GET; Supplementary Data Table S1) and two populations of the P+ phenotype (P+_ACA, P+_BNS). Each of the four combinations of crosses was replicated three times (i.e. there were 12 crossing arrays in total). Arrays were established as squares of 49 plants, with 25 P– and 24 P+ individuals in each array, arranged in spatial alternation. Plants were harvested after 5 weeks of growth and mating in their array, and 30 progeny from the set of P– or P+ mothers per array were raised on a glasshouse bench in a complete randomized block design. For each progeny individual, we recorded above-ground biomass, and female and male reproductive efforts. We determined the paternity for each progeny individual (i.e. whether it had been sired by a P+ or P– individual) on the basis of four microsatellite markers (see below).

To assess the male fertility of hybrids between P+ and P– parents, we first checked the morphology of anthers under a stereomicroscope (Wild M3, Heerbrugg, Switzerland), and counted the number of anthers, number of pollen grains and pollen size using a particle size analyser (Micromeritics Elzone II, Norcross, GA, USA). Because different lineages of *M. annua* are able to pollinate and fertilize one another, including between ploidy levels, we further assayed the siring potential of the *F*_1_ progeny by allowing them to pollinate the stigmas of diploid *M. annua* females and assaying their siring success; we performed this assay on diploid females rather than hexaploid monoecious individuals to avoid the complication of self-pollination. In total, we established three isolated mating ‘triplets’, with each triplet comprising one *F*_1_ (progeny of P– and P+) hybrid individual together with two diploid females; the triplet crosses were established individually in one of three separated pollen-proof incubators (~23 °C, 50 % humidity, 12: 12-h light–dark cycle). We also established three replicate crosses between diploid *M. annua* females and males as a control to ensure that culture conditions allowed effective crossing under similar conditions in a glasshouse.

### Paternity assignment for progeny from crossing arrays

For DNA extraction, the fresh leaf material was first dried in an oven at 40 °C for 3 d. The dry leaves (10–15 mg) were then ground to a powder with beads using a TissueLyser II machine (Qiagen, Valencia, CA, USA) for subsequent DNA extraction, following the Biosprint 96 protocol provided by the manufacture. All plant tissue samples were then processed using the BioSprint 96 workstation with the BS 96DNA Plant program, resulting in 200 µL DNA in elution buffer (Qiagen).

We determined the paternity class of each progeny on the basis of population-specific microsatellite markers developed previously in the lab (primer sequences and PCR conditions are described in Korbecka *et al.*, 2011). We used these microsatellites to distinguish between siring by other P– or P+ individuals in each array, as well as to estimate average selfing rates. We genotyped 30 plants from progeny produced by each of the two types of monoecious mothers from each of the 12 replicate arrays (a total of 720 plants). Individuals were scored for each of four microsatellite markers (Mh14, Mh15, Mh19, Mh52, Supplementary Data Table S2) that are known to provide good separation of the hexaploid populations sampled (Korbecka *et al.*, 2010). All four markers were amplified in a single multiplexed PCR, following the protocol described in Korbecka *et al.* (2010). We processed the samples in an ABI 3100 sequencer (Applied Biosystems, Carlsbad, CA, USA), and analysed the results with GeneMapper v.4.0 (Applied Biosystems). Individual genotypes were classified as having been sired by a father of the same or the competing phenotype in the array. We estimated selfing rates using the software RMES (David *et al.*, 2007). This was only possible for the populations of P– individuals, as populations with P+ individuals had almost no genetic variability for the markers used (Supplementary Data Table S2); for P+ individuals we also distinguished between P– and P+ sires, but could not estimate selfing rates. This limitation did not affect the interpretation of *F*_1_ progeny between P+ and P– individuals.

### Sanger sequencing and phylogeny construction

To investigate the phylogenetic relationship between P– and P+ individuals in our samples and with other lineages in the genus, we constructed phylograms on the basis of the same loci as those used for phylogenetic reconstruction of the genus by Obbard *et al.* (2006a), i.e. two chloroplast markers, *trnL-trnF* and *matK-trnK*, as well as the nuclear internal transcribed spacer 2 (*ITS2*). For each individual sampled, we collected fresh leaves, dried them and performed DNA extraction as described above. DNA quality of leaves from two P– populations was insufficient for genotyping (due to poor plant health prior to sampling), and we excluded these populations from the following phylogenetic analysis described below. We used cloning to identify potentially different copies of *ITS2* in the hexaploid genome. Because cloning can separate individual sequence copies, sampling a number of colonies can potentially allow identification of all *ITS2* copies for a particular genome. Cloning was performed using the Promega pGEM-T Easy Vector System II, following the manufacturer’s protocol. The PCR and cloning products were sequenced by Microsynth (Balgach, Switzerland). Raw sequences were trimmed with Geneous v.2 (http://www.geneious.com;Kearse *et al.*, 2012), and ambiguous nucleotides were checked by eye and corrected manually. After trimming, sequences of all individuals were the same length for a given locus: *ITS2*, *trnL-trnF* and *matK-trnK* sequences were 679, 415 and 560 bp in length, respectively. All primer sequences as well as the protocol for PCRs are described in Obbard *et al.* (2006a).

We used MrBayes (v.3.2.6) for phylogenetic reconstruction (Ronquist *et al.*, 2012), combining our own sequences for nine P– and 11 P+ populations with those downloaded from the NCBI database (populations from the same and other ploidy levels of *M. annua*, as described and analysed in Obbard *et al.* (2006a). MrBayes uses Bayesian inferences and Markov chain Monte Carlo (MCMC) methods to estimate the posterior distribution of model parameters. First, sequences for each marker were aligned using the multiple sequence alignment function in ClustalW (Larkin *et al.*, 2007). For the aligned sequences of each marker, the best partition scheme and nucleotide substitution model were selected using PartitionFinder (Lanfear *et al.*, 2012). Aligned sequences were analysed in MrBayes, seeking the best partition scheme and substitution model parameters with an MCMC chain of 10 million iterations. The trace files generated by Bayesian MCMC runs were verified using Tracer software until all parameter values sampled from the chain reached their optima (http://tree.bio.ed.ac.uk/software/tracer/). Finally, the generated tree file was visualized in FigTree software (http://tree.bio.ed.ac.uk/software/figtree/).

## RESULTS

### Morphological trait differences between inflorescence phenotypes

**Fig. 2.**
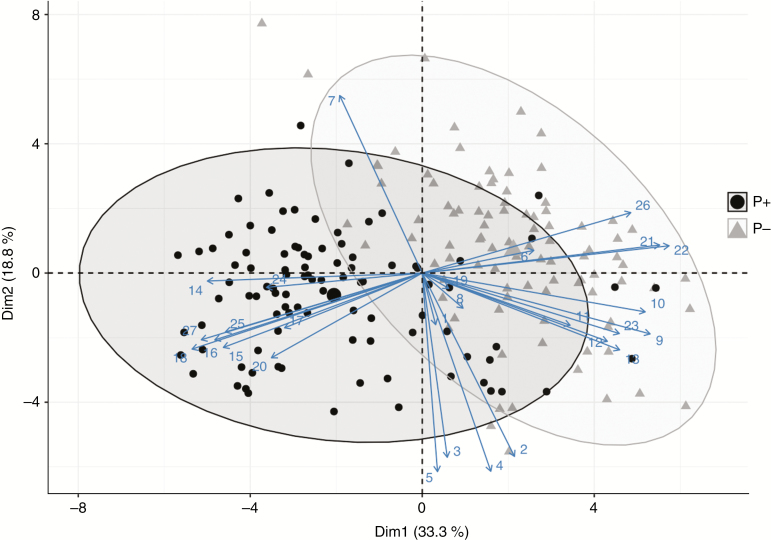
Principal component plot of multiple morphological traits for P– and P+ monoecious hexaploid individuals of *Mercurialis annua*. Solid black circles and solid grey triangles denote P+ and P– populations, respectively. Numbers refer to traits as follows: 1, petiole length; 2, leaf blade length; 3, leaf blade width; 4, leaf blade area; 5, leaf blade perimeter; 6, leaf blade circularity; 7, leaf blade perimeter to area ratio; 8, leaf blade to petiole ratio; 9, plant height; 10, average branch length; 11, length of 3rd internode; 12, length of 4th internode; 13, length of 5th internode; 14, presence of curly leaves; 15, number of peduncles; 16, numbers of apical peduncles; 17, number of peduncles with fruits; 18, average peduncle length; 19, presence of trichomes on stems; 20, pollen biomass; 21, seed biomass; 22, seed number; 23, above-ground biomass; 24, individual seed weight; 25, pollen allocation; 26, seed allocation; 27, sex allocation.

Most vegetative traits differed significantly between the P– and P+ phenotypes, but there was also substantial variation among populations within each phenotype (Table 1). Overall, PCA separated the two phenotypes into two clusters, albeit with some overlap (Fig. 2). Although most leaf traits (i.e. leaf blade area, leaf blade perimeter, leaf blade width, leaf petiole length and leaf perimeter area) did not differ between the two phenotypes, the leaf margins of the P+ phenotype were significantly more undulating or ‘curly’ than those of the P– phenotype (Table 1). The first and second PCA axes accounted for 33.3 and 18.8 %, respectively, of the overall morphological variation (Fig. 2).

**Table 1.** Measures of morphological traits for the two distinctive monoecious phenotypes

Trait	P– (mean ± s.d.)	P+ (mean ± s.d.)	*P* value	Bonferroni-corrected *P* value
Leaf blade area (mm^2^)	1410 ± 434	1460 ± 299	0.54	1
Leaf blade circularity	0.38 ± 0.04	0.35 ± 0.04	**0.03**	0.9
Leaf blade length (mm)	52.40 ± 8.90	52.60 ± 6.20	**0.02**	0.6
Leaf blade perimeter (mm)	212.00 ± 35.40	226.90 ± 22.30	0.84	1
Leaf blade width (mm)	33.20 ± 5.60	34.90 ± 4.10	0.09	1
Blade to petiole length ratio	3.47 ± 1.18	3.56 ± 1.28	0.90	1
Leaf petiole length (mm)	17.30 ± 5.00	16.70 ± 4.30	0.67	1
Leaf perimeter to area ratio	0.014 ± 0.003	0.014 ± 0.02	0.95	1
Proportion of plants with curly leaves	0.06 ± 0.05	0.71 ± 0.10	**<0.001**	**0.003**
Above-ground biomass (g)	4.33 ± 0.98	3.8 ± 0.91	**0.02**	0.6
Length of branch 0 (cm)	17.50 ± 5.97	12.90 ± 4.82	0.24	1
Length of branch 1 (cm)	15.40 ± 5.63	12.00 ± 3.99	**0.03**	0.9
Length of branch 2 (cm)	15.70 ± 5.68	12.10 ± 4.71	**0.05**	1
Plant height (cm)	30.40 ± 7.77	23.10 ± 6.35	**0.01**	0.3
Length of 4th internode (mm)	44.40 ± 11.90	36.10 ± 10.70	**0.04**	1
Length of 5th internode (mm)	44.10 ± 13.80	35.20 ± 12.40	**0.05**	1
Length of 3rd internode (mm)	40.10± 9.43	31.90 ± 8.48	**0.01**	0.3
Proportion of plants with trichomes	0.11 ± 0.10	0.03 ± 0.04	0.19	1
Average number of clusters of male flowers	0.94 ± 0.75	1.92 ± 0.76	**<0.001**	**0.003**
Length of peduncle (mm)	7.4 ± 8.89	54.80 ± 23.60	**<0.001**	**0.003**
Number of peduncles with fruits	0.59 ± 1.85	11.37 ± 9.58	**<0.001**	**0.003**
Number of peduncles	1.36 ± 4.03	32.90 ± 20.90	**<0.001**	**0.003**
Number of apical peduncles	0.18 ± 0.8	7 ± 10.90	**<0.001**	**0.003**
Male reproductive effort	26.60 ± 16.02	47.40 ± 33.70	**<0.001**	**0.003**
Pollen biomass (mg)	117.0 ± 62.70	188.20± 91.70	**<0.001**	**0.003**
Seed biomass (g)	2.31 ± 0.28	2.66 ± 0.48	**0.01**	0.3
Seed number	526.80 ± 222.60	285.20 ± 190.10	**<0.001**	**0.003**
Female reproductive effort	120.20 ± 41.90	71.80 ± 38.40	**<0.001**	**0.003**
Seed size (µm)	231.50 ± 102.10	113.10 ± 87.70	**<0.001**	**0.003**
Sex allocation	0.19 ± 0.12	0.4 ± 0.25	**<0.001**	**0.003**

P values are derived from generalized linear mixed models (GLMMs), and bonferroni adjusted P values were provided for correcting multiple test. P values < 0.05 are in bold.

**Fig. 3.**
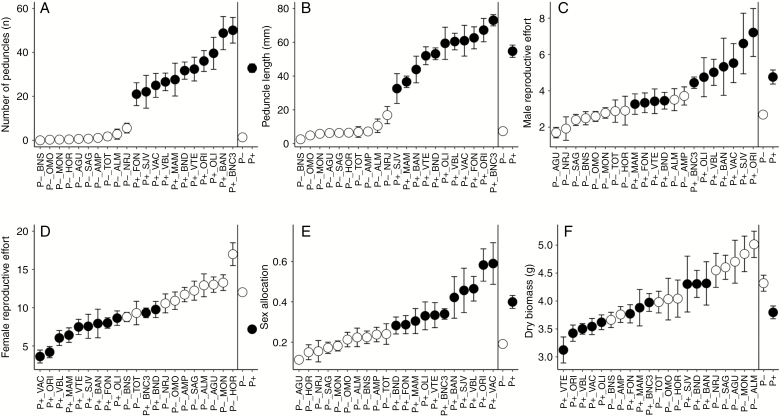
Morphological trait measurements (mean and standard error) of P+ and P– monoecious hexaploid populations of *Mercurialis annua* from the common-garden experiment: (A) number of peduncles, (B) peduncle length, (C) male reproductive effort, (D) female reproductive effort, (E) sex allocation, and (F) above-ground biomass. Grey and black circles denote P – and P+ populations, respectively.

The P+ phenotype had ~25 times more peduncles than the P– phenotype (*P* < 0.0001, Fig. 3A), and peduncles on P+ plants were overall seven times longer than those on P– plants (*P* < 0.0001, Fig. 3B). Plants of the P+ phenotype also invested significantly more resources toward male function (in terms of male reproductive effort) than did those of the P– phenotype (*P* < 0.0001, Fig. 3C), and correspondingly fewer resources to female function (in terms or female reproductive effort; *P* < 0.0001, Fig. 3D). Accordingly, the sex allocation of the P+ phenotype was skewed substantially towards the male function relative to the P– phenotype (Fig. 3E), although the P+ phenotype had overall significantly lower above-ground biomass than the P– phenotype (*P* < 0.0001, Fig. 3F).

### Differences in DNA content between inflorescence phenotypes

**Fig. 4.**
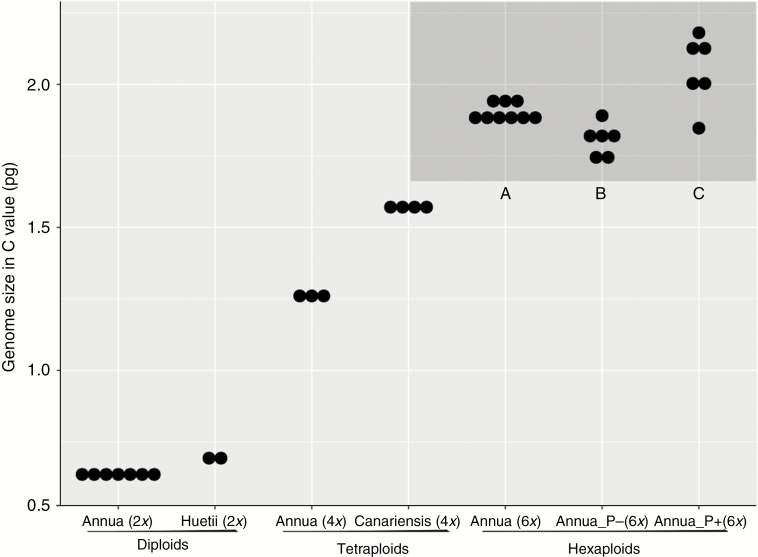
Mean genome size (measured as C value of DNA content, in pg) of different *Mercurialis* lineages, with ploidy levels indicated below each species. Each solid dot is the mean value of several individuals from one population. Data from *Mercurialis* lineages are from Obbard *et al.* (2006a). The grey area indicates DNA content of hexaploid lineages, and A, B and C denote significance at P < 0.001 for the corresponding pairwise comparisons.

Flow cytometry confirmed that both P– and P+ phenotypes are probably hexaploid, i.e. DNA content corresponds roughly to that observed for hexaploid *M. annua* previously (Obbard *et al.*, 2006a), and was about three times larger than for diploids of the species complex. However, we nonetheless found significant variation in genome size among populations within each lineage assayed, particularly for the hexaploid populations (Fig. 4). Specifically, individuals of the P+ phenotype had somewhat higher DNA content (2.05 ± 0.055 pg) than those of the P– phenotype (1.81 ± 0.025 pg), and varied more among populations (*P* < 0.001, Fig. 4).

### Reciprocal crossing experiment, hybrid viability and hybrid sterility

**Fig. 5.**
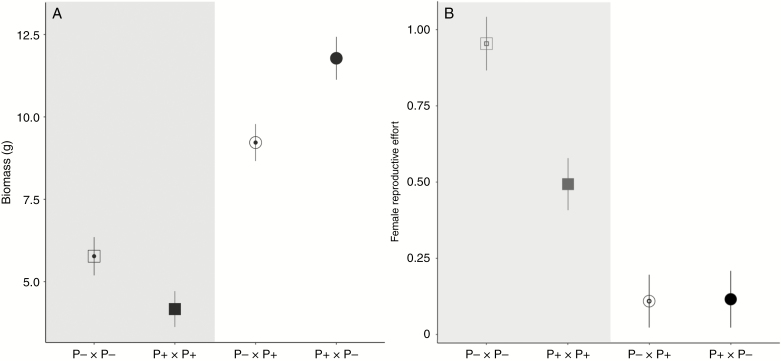
(A) Above-ground biomass and (B) female reproductive effort for offspring from control crosses (in the grey area, square symbol) and reciprocal crosses (round symbol) between individuals with female-like inflorescence morphology (P–) and male-like inflorescence morphology (P+). The maternal parent is indicated first in each cross type, with symbols for P– and P+ being open and closed, respectively.

Progeny of crosses between P+ and P– individuals were fully viable, and indeed the above-ground biomass of *F*_1_ hybrids was two- to three-fold higher than that of progeny resulting from crosses within each phenotype (*P* < 0.001, Fig. 5A; Supplementary Data Table S2). However, *F*_1_ hybrids were largely female-sterile, regardless of the direction of the cross, with very few viable seeds produced (most seeds were aborted before they reached their mature size; *P* < 0.001, Fig. 5B). We were unable to compare the siring ability of the *F*_1_ individuals in a fully balanced experiment, but it would seem that the male fertility of *F*_1_ hybrids is probably also impaired: although *F*_1_ individuals produced similar numbers of anthers and pollen grains to their parents (see Supplementary Data Fig. S2A–F), they sired only two seeds on diploid females in a growth chamber, whereas hexaploid hermaphrodites from either parental P– or P+ populations sired a large number of seeds under broadly similar conditions in a glasshouse.

### Phylogenetic reconstruction

#### Plastid sequences

**Fig. 6.**
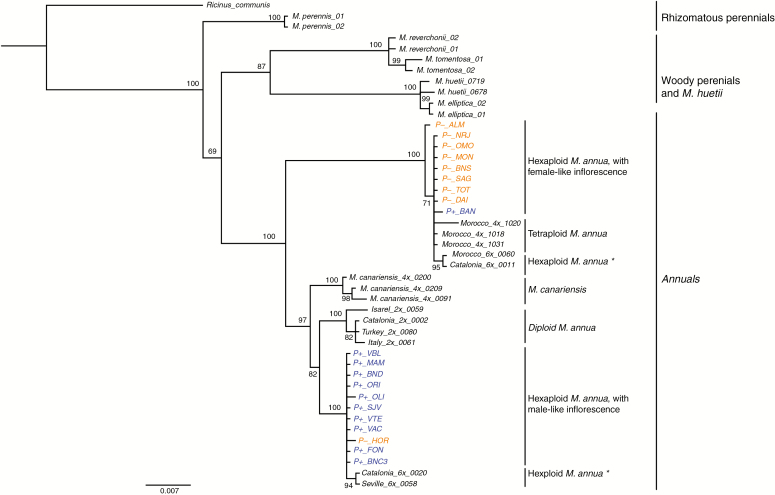
MrBayes consensus tree based on the concatenated *trnL-trnF* and *matK-trnK* chloroplast datasets, using the best-fit partition model of nucleotide substitution with 10 million MCMC burn-in iterations. P– and P+ monoecious populations are highlighted in orange and blue, respectively. Asterisks denote hexaploid *Mercurialis annua* populations for which inflorescence structure was not measured. The scale bar at the bottom are in the units of number of base substitutions per site.

Phylogenetic analysis of a concatenation of *trnL-trnF* and *matK-trnK* chloroplast sequences strongly supports a clade that includes all but one of the P– monoecious populations. Within this clade, all individuals from populations characterized by the P– morphology were clustered with those from tetraploid populations of *M. annua*, as well as those from two hexaploid populations from Morocco and Catalonia (Fig. 6). All but one of the P+ monoecious individuals formed a separate clade, which included those of two other hexaploid *M. annua* populations from Seville and from Catalonia, as well as from diploid populations of *M. annua* and tetraploid *M. canariensis*. The exceptional individual from the female-like monoecious population P-_HOR falls into the largely P+ clade, while the exceptional individual from the male-like monoecious population P+_BAN falls into a clade with individuals of the P– phenotype.

#### Nuclear sequences

**Fig. 7.**
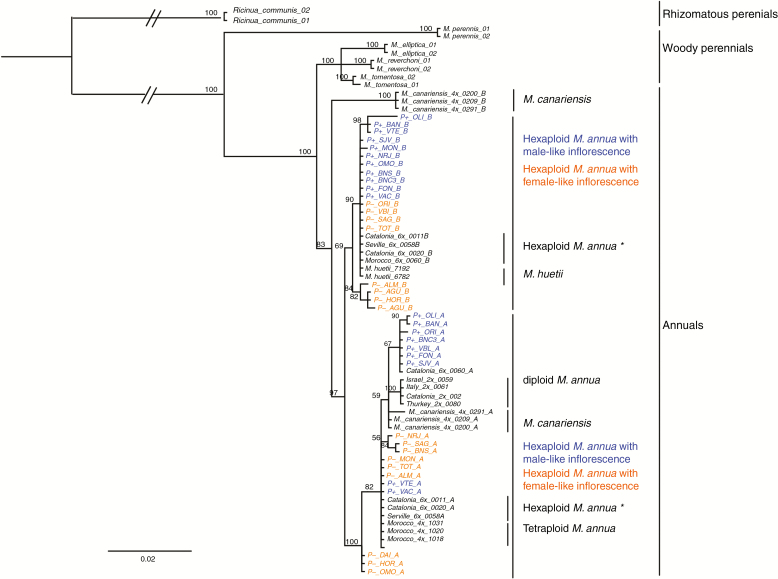
MrBayes consensus tree based on the *ITS* datasets, using the best-fit partition model of nucleotide substitution with 10 million MCMC burn-in iterations. P– and P+ monoecious populations are highlighted in orange and blue, respectively. The scale bar at the bottom are in the units of number of base substitutions per site.

Given that all P+ and P– individuals sampled were probably hexaploid, they should each have more than one *ITS2* sequence copy. Indeed, phylogenetic reconstruction based on the nuclear marker *ITS2* points to an allopolyploid hybrid origin for the hexaploid lineages, with all P+ or P– individuals having *ITS2* copies from both diploid and tetraploid *M. annua*, on the one hand, and diploid *M. huetii*, on the other (Fig. 7). This result confirms the findings of Obbard *et al.* (2006a) for hexaploid *M. annua* generally. Furthermore, within the clade of sequences representing the diploid *M. annua ITS*2 copy, phylogenetic analysis grouped together individuals of tetraploid *M. canariensis*, tetraploid *M. annua*, and all hexaploid *M. annua* populations, including those with both P+ and P– morphology. All hexaploid individuals of *M. annua* fell into a clade with *M. huetii*, which excluded individuals of *M. canariensi*s and tetraploid *M. annua* (Fig. 7).

## DISCUSSION

### *Origin and taxonomy of a new biological species in the* M. annua *species complex*

Three lines of evidence suggest that individuals of *M. annua* with the P+ phenotype belong to a different biological species from those with the more widely distributed P– phenotype, which occurs either in purely monoecious or, together with males, in androdioecious populations: (1) individuals of the P+ lineage are morphologically distinct from those of the P– lineage; (2) *F*_1_ progeny produced in crosses between the two phenotypes are largely (but not completely) female-sterile and show signs of male sterility, too; and (3) individuals of the two phenotypes fall largely into separate clades of a chloroplast-based gene tree. We also found that *F*_1_ progeny from crosses between the two phenotypes were substantially larger than the non-hybrid progeny. This result may point to heterosis or hybrid vigour, consistent with both parental lineages being inbred (Semel *et al.*, 2006; Hochholdinger and Hoecker, 2007; Wang *et al.*, 2015), and as suggested by the low genetic diversity observed in P+ populations (Santos del Blanco *et al.*, 2019), but also in many P– populations (Obbard *et al.*, 2006b; Pujol *et al.*, 2009, 2010). Alternatively, it is possible that some of the vigour of hybrid individuals is attributable to trade-offs resulting from their lower reproductive effort.

Both biological species of *Mercurialis* studied here appear to be hexaploid hybrids, probably allohexaploids generated from a cross between *M. annua* and *M. huetii.* In contrast to the chloroplast gene tree, which separates the two hexaploid lineages, the nuclear gene tree fails to group them into different clades. This pattern might be the result of incomplete lineage sorting, or of recent or ongoing gene flow between the two lineages. In the absence of further sampling, it is not possible to distinguish between these two possibilities (Maddison and Knowles, 2006; Joly *et al.*, 2009; Zhou *et al.*, 2017). However, incomplete lineage sorting appears to be a less likely explanation if both lineages arose through a narrow bottleneck, as expected for new polyploids (e.g. Willyard *et al.*, 2009). The possibility of ongoing gene flow would be consistent with the current broad sympatry of the two lineages.

Polyploidization and hybridization have played an important role in the diversification of the annual clade of *Mercurialis* (Obbard *et al.*, 2006a), as they have for flowering plants in general (Pannell *et al.*, 2004; Adams and Wendel, 2005; Otto, 2007; Soltis *et al.*, 2014b). Diploid *M. annua* and its diploid sister species *M. huetii* have previously been hypothesized as the original parents of two tetraploid lineages (monoecious *M. annua*, currently found in south-western Morocco; and dioecious *M. canariensis*, currently endemic to the Canary Islands), as well as one hexaploid lineage (androdioecious *M. annua*, i.e. the P– lineage, widespread in northern Morocco and around the Iberian Peninsula) (Obbard *et al.*, 2006b). The two diploid *Mercurialis* species hybridize easily, but hybrid progeny suffer high sterility in both their male and their female functions (Russell and Pannell, 2015). The present study now suggests that the P+ hexaploid lineage, comprising the morphologically and functionally distinct male-like monoecious individuals, has also been generated by genome duplication and hybridization. The remarkable feature of this process of diversification in *Mercurialis* is that both pairs of independently derived polyploid lineages (the two tetraploid and the two hexaploid lineages) have contrasting sexual systems: tetraploids that are either dioecious or monoecious; and hexaploids that are either androdioecious (males coexisting with female-like P– monoecious individuals), monoecious P+ (individuals with their distinct male-like inflorescence) or monoecious P– (populations with female-like P– monoecious individuals without males).

**Fig. 8.**
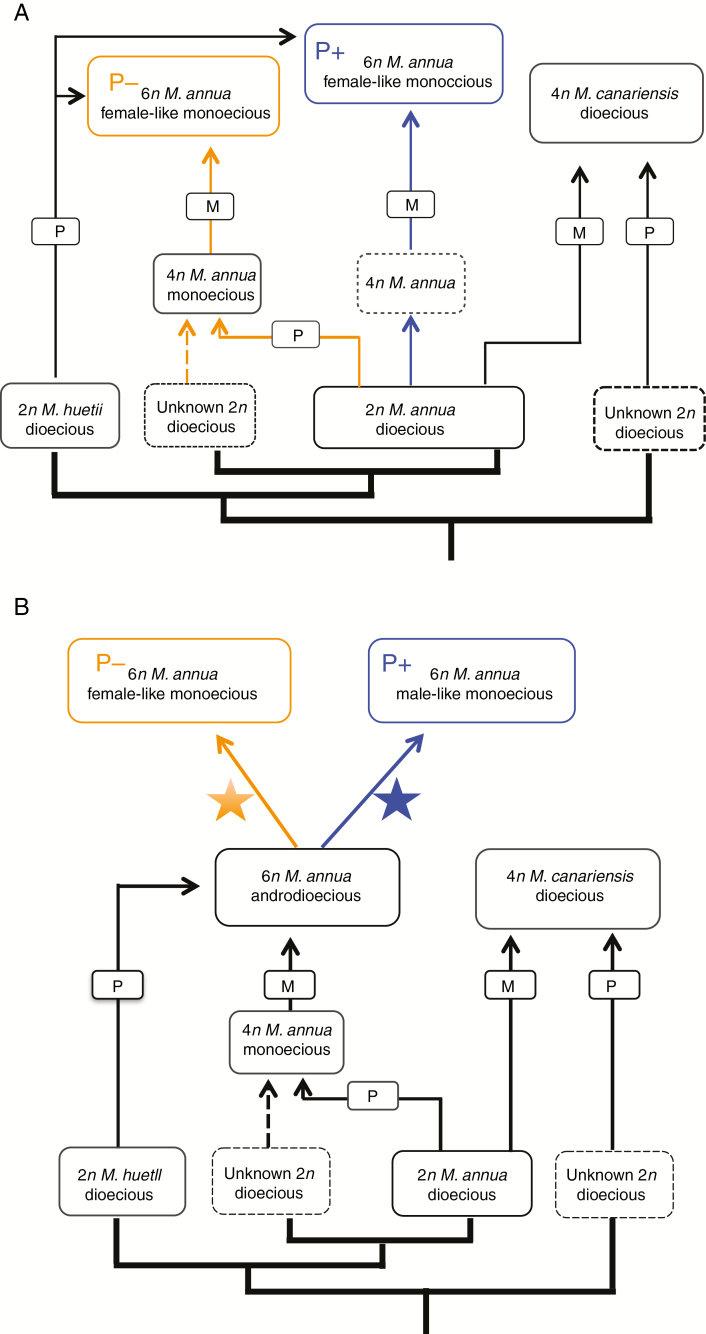
Modified scheme of two possible scenarios for the evolutionary origin of the P+ hexaploid monoecious phenotype within the *Mercurialis* species complex. Scenario A describes two independent evolutionary origins of P+ and P– hexaploid monoecious phenotypes. Here, the P+ hexaploid phenotype is derived from a possible autopolyploidization of diploid dioecious *M. annua* to form an unknown tetraploid *Mercurialis* species (indicated with blue lines), followed by hybridization with a diploid *M. huetii* male. The P– hexaploid lineage is then possibly derived from hybridization between an unknown maternal diploid *Mercurialis* species and a diploid *M. annua* male, followed by autopolyploidization to form tetraploid *M. annua* (orange lines), finally followed by hybridization with a diploid *M. huetii* male. Scenario B depicts a single origin for both P+ and P– phenotypes. Here, the evolutionary origin of hexaploid androdioecious *M. annua* follows the hypothesis of Obbard *et al.* (2006a). Both P– and P+ evolved from hexaploid androdioecious *M. annua*, during which reproductive isolation evolved, with chloroplast capture between different hexaploid lineages or between hexaploid and diploid *M. annua*. Figure modified from Obbard *et al.* (2006a). A star associated with orange or blue denotes a different diversifying process.

Further sampling, both of genotypes and of more nuclear loci, will be required to establish with more confidence the evolutionary paths that led to the origin of the two different hexaploid lineages. Figure 8 recapitulates the scheme proposed by Obbard *et al.*, (2006a) for the relationships between the diploid, the tetraploid and the P– hexaploid lineages of the annual mercuries, modified with hypotheses for the origin of the P+ lineage. At least two scenarios seem plausible. One scenario is that the P– and P+ lineages are the result of two independent polyploid hybridization events involving ancestors of the current diploid dioecious lineages *M. huetii* and *M. annua* (Fig. 8A). The other scenario is that both lineages are the result of a single polyploid hybridization event that generated an ancestral hexaploid lineage, and that this population subsequently diversified into the two separate P– and P+ lineages (Fig. 8B). The current overlapping geographical distributions of these two lineages in south-eastern Spain would seem to argue more against the latter scenario, but it is plausible that the two lineages originated or diverged in allopatry and only subsequently came to occupy their current range, with occasional recent gene flow between them explaining the observed sharing of ITS variation.

Regarding the origin of the two hexaploid lineages, it does not seem possible to attribute much significance to their association with distinct chloroplast haplotypes; this pattern might simply be the result of differential chloroplast capture from a related lineage subsequent to divergence. Chloroplast capture, which results from hybridization between lineages with different chloroplast haplotypes and subsequent introgression by repeated asymmetrical backcrossing (Jackson *et al.*, 1999; Tsitrone *et al.*, 2003; Acosta and Premoli, 2010), has been common in angiosperm evolution and is a probable cause of incongruence between chloroplast gene trees and species trees in many phylogenies (Mort *et al.*, 2002; Fehrer *et al.*, 2007; Baldwin *et al.*, 2011; Dong *et al.*, 2012). Indeed, *M. huetii* itself has a chloroplast that is divergent from that of its sister species *M. annua*, and that probably introgressed from perennial species of the genus (Obbard *et al.*, 2006a). Thus, although a more complete picture of the evolutionary origin of the hexaploid phenotypes of *M. annua* will require analysis of more markers across the genome, the current limited sampling of both chloroplast and nuclear markers already points to a complex history involving genome duplication, hybridization and potentially on-going gene flow and introgression among diverging lineages. It is of course also possible that the P+ population in the P– clade is the result of convergent evolution.

The taxonomy and systematics of the genus *Mercurialis* are clearly in need of revision. Several different names refer to the various annual lineages of *Mercurialis*. The two diploid dioecious lineages, *M. annua* and *M. huetii*, would appear to be species with acceptable names; although having the same ploidy, these species are morphologically and genetically distinct, and their hybrids are largely sterile (although viable). The nomenclature of the tetraploid and hexaploid lineages that probably descend from ancestors of *M. annua* and *M. huetii*, however, is inconsistent and problematic. These lineages have variously been grouped together under the same label as *M. annua* or *M. ambigua*. The epithet *ambigua* unifies tetraploid and hexaploid lineages that are morphologically difficult to distinguish (Obbard *et al.*, 2006a), but that are reproductively isolated from one another (Russell and Pannell, 2015). By contrast, the epithet *annua* unifies dioecious diploid *M. annua* with largely monoecious tetraploid and hexaploid lineages, i.e. includes one of their putative diploid parents (*M. annua*) with the tetraploid and hexaploid lineages, but excludes the other putative progenitor (*M. huetii*) as well as tetraploid dioecious *M. canariensis*. Moreover, both *annua* and *ambigua* also exclude lineages with higher ploidy levels that occur in North Africa, Corsica and Sardinia, which have been sometimes referred to as *M. monoica* (Durand, 1963; Pannell, 1997*b*; Pannell *et al.*, 2004). The current study now reveals that the hexaploid populations of *M. annua* (*sensu lato*) comprise at least two different lineages that are morphologically more distinct from one another than is the common P– phenotype from tetraploid *M. annua* (*sensu lato*) (Obbard *et al.*, 2006a). Although it may have been convenient to refer loosely to the *M. annua* species complex with the exclusion of *M. huetii*, a comprehensive revision of the clade would now be useful, not least because the lineages referred to are both reproductively isolated from one another and morphologically and functionally distinct.

### *Maintenance of ploidy and reproductive diversity in the* M. annua *species complex*

Although the different lineages of the *M. annua* species complex, including *M. huetii*, differ in their sexual systems, they occupy very similar ruderal and roadside habitats. How then are they being maintained? This question is particularly pressing for lineages on the Iberian Peninsula that are in broad sympatry with one another, not least because they are mobile colonizers of disturbed habitats. The problem of coexistence applies particularly to the newly characterized P+ phenotype, but it is general to the whole species complex. For example, in both north-eastern and north-western Spain, diploid dioecious *M. annua* is parapatric to hexaploid *M. annua*, whose populations are almost exclusively monoecious at the zones of contact. Here, diploid individuals appear to be physiologically superior to their hexaploid counterparts, but are also displacing them as a result of asymmetrical pollen swamping and hybridization that results in sterile progeny (Buggs and Pannell, 2006). Indeed, historical data indicate that this demographic displacement is occurring at a rate of several kilometres per year in both zones of contact and might be expected to lead to the ultimate of demise of P– hexaploid *M. annua* entirely, as a result of diploid expansion (Buggs and Pannell, 2006).

Given the superiority in both pollen production and dispersal ability of the P+ over the P– hexaploid hermaphrodites (Santos del Blanco *et al.*, 2019), we might expect that, over time, the P+ phenotype will eventually displace the P– phenotype as a result of pollen swamping similar to that experienced by the P– lineage when it encounters dioecious diploid *M. annua* (Buggs and Pannell, 2006). Indeed, in the common garden experiment in which P+ and P– individuals were grown together at equal frequencies, the great majority of seeds produced by both phenotypes were sired by P+ individuals (Supplementary Data Table S2). We speculate that displacement of the P– by the P+ lineage should be even more rapid than by diploids, because only half of all diploid individuals (i.e. the males) produce pollen. Determining whether the P+ lineage could resist displacement by diploids, or could even displace the diploids themselves, will require array experiments similar to those conducted by Buggs and Pannell (2006) and Santos del Blanco *et al.* (2019).

A plausible alternative scenario to the demographic displacement of P– by the P+ phenotype through pollen swamping is the selective introgression into the genomic background of the P– lineage of genes responsible for the pedunculate inflorescence. As discussed above, there is already some evidence for this possibility from the genotypes observed in our small sample in this study: one individual with a male-like inflorescence had a chloroplast haplotype more typically associated with the P– lineage (and vice versa). Given the much higher siring success, and probably greater fitness, of individuals with male-like inflorescences (Santos del Blanco *et al.*, 2019), the selective introgression of the genes responsible would seem highly likely, and it is surprising that the male-like inflorescence has not already swept through the range of the P– phenotype. Such selective introgression is well known for other plant traits. For instance, C_4_ photosynthesis that is advantageous under conditions of high insolation, high temperature and drought is known to have introgressed into formerly C_3_ plants that had presumably moved into environments in which C_4_ photosynthesis was favoured (Christin *et al.*, 2008; Gowik and Westhoff, 2011). Similarly, advantageous mutations altering that function of the key protein involved in photosynthesis, Rubisco, appear to have moved among species of the genus *Schiedea* by selective introgression (Kapralov *et al.*, 2011). Other similar examples of selective introgression have been recorded in animals (Albertson *et al.*, 2003; Quinn *et al.*, 2007).

### Independent origins of polyploidy as a further engine for diversification?

The *M. annua* species complex is remarkable for its sexual-system variation, but it is wholly unremarkable in terms of its ploidy variation: within-species variation in ploidy is a hallmark of many plant species (Lewis, 1980; Soltis *et al.*, 2007; Beest *et al.*, 2012). Indeed, genome duplication has contributed substantially to plant diversification in general (Stebbins, 1950; Levin, 1983; Soltis *et al.*, 2014a), probably both by expanding or shifting the environmental niches that new polyploid populations can occupy (Beest *et al.*, 2012), but also by establishing instantaneous reproductive isolation between a newly derived polyploid population and its lower-ploidy progenitor species (Ramsey and Schemske, 1998; Seehausen, 2004; Husband and Sabara, 2011). Our study indicates that populations with the same ploidy level but with different origins may also be reproductively isolated from one another, although the breakdown of homoploid reproductive barriers with rising ploidy is known for some lineages (Ehrendorfer, 1996; Lafon-Placette *et al.*, 2017).

To the extent that rapid genetic changes immediately following genome duplication and/or hybridization associated with polyploidy should occur independently in lineages with independent origins, our conclusion should not be so surprising – although the polyphyletic origins of one and the same ploidy level are perhaps an underappreciated engine of plant diversification. In the case of *M. annua* hexaploids, the occupancy of P– and P+ lineages of very similar habitats and their coincident flowering phenologies are likely to bring about the displacement of one lineage by the other due to pollen swamping (just as the P– lineage is currently being displaced by the diploid lineages in northern Spain; Buggs and Pannell, 2006), diminishing the likelihood of a net positive effect on diversification. However, a scenario of multiple origins of polyploidy might have a more positive effect on long-term diversification when divergent populations are maintained in allopatry by occupying different ecological niches, or when reproductive interference is avoided through the evolution of prezygotic isolation. Such a scenario would seem to be more likely in animal- than in wind-pollinated species (e.g. Moyle *et al.*, 2004; Rieseberg *et al.*, 2006; Rieseberg and Willis, 2007; Lowry *et al.*, 2008).

## SUPPLEMENTARY DATA

Supplementary data are available online at https://academic.oup.com/aob and consist of the following. Fig. S1: Sampling locations for both P– and P+ monoecious hexaploid populations of *M. annua* along the east coast of the Iberian Peninsula. Fig. S2: Comparison of male flowers between *F*_1_ hybrids of P+ and P– lineages and hexaploid monoecious individuals and between these *F*_1_ hybrids and hexaploid males. Table S1: Sampled populations, their original location, abbreviation and the experiments carried out in this study. Table S2: Source populations for reciprocal crosses between P+ and P– phenotypes, and the genotypes of four microsatellite markers.

mcz058_Suppl_Supplementary_Figure_S1

mcz058_Suppl_Supplementary_Figure_S2

mcz058_Suppl_Supplementary_Table_S1

mcz058_Suppl_Supplementary_Table_S2

## FUNDING

This work was supported by the Sinergia grant CRSII3_147625 from the Swiss National Science Foundation to J.R.P., Nicolas Perrin and Mark Kirkpatrick.
