# Supplementary figures and images for: A new biological species in the Mercurialis annua polyploid complex: functional divergence in inflorescence morphology and hybrid sterility
Source: Ann Bot. 2019 May 17;124(1):165–78. doi: 10.1093/aob/mcz058 (PMC6676388; doi:10.1093/aob/mcz058)

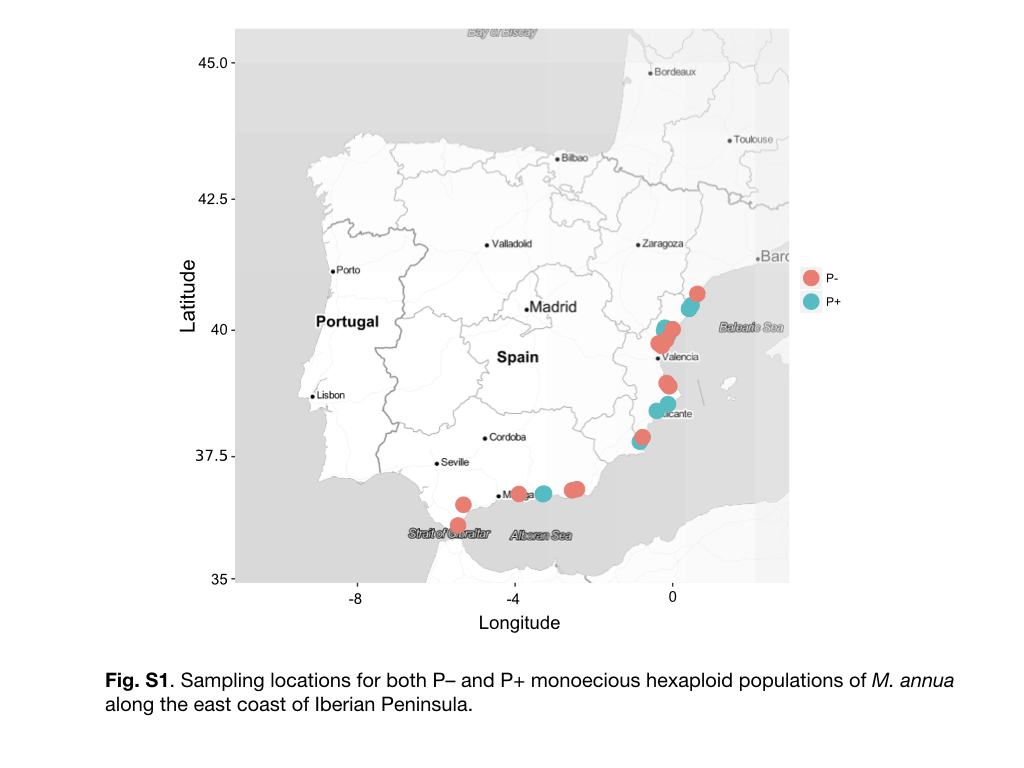

Supplement: mcz058_Suppl_Supplementary_Figure_S1 [file mcz058_suppl_supplementary_figure_s1.png]

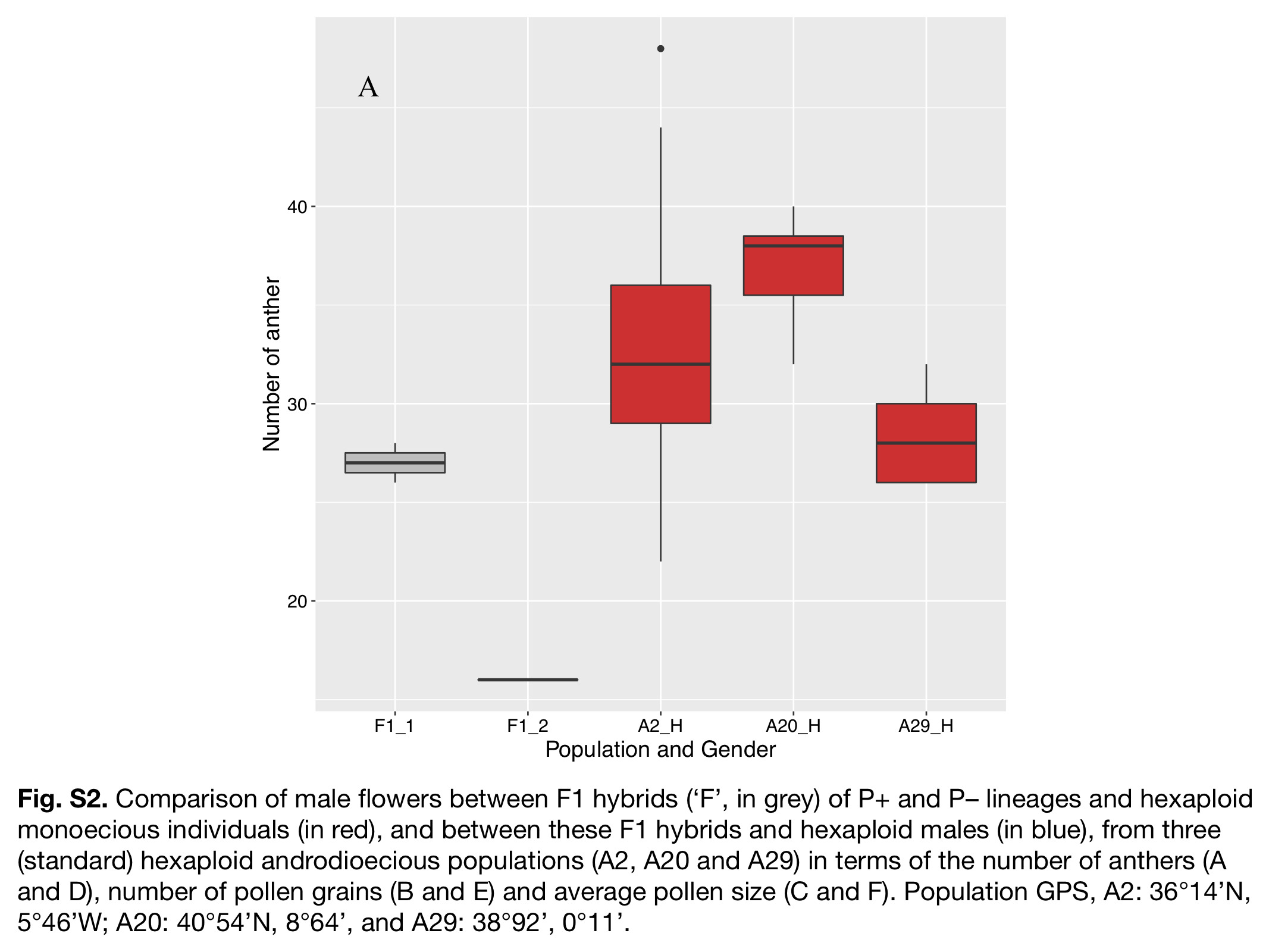

Supplement: mcz058_Suppl_Supplementary_Figure_S2 [file mcz058_suppl_supplementary_figure_s2.jpeg]
